# Robust vaccine formulation produced by assembling a hybrid coating of polyethyleneimine–silica†
†Electronic supplementary information (ESI) available: Detailed experimental information, and some experimental results. See DOI: 10.1039/c5sc03847b



**DOI:** 10.1039/c5sc03847b

**Published:** 2015-12-10

**Authors:** Guangchuan Wang, Hangyu Zhou, Qing-Gong Nian, Yuling Yang, Cheng-Feng Qin, Ruikang Tang

**Affiliations:** a Qiushi Academy for Advanced Studies , Zhejiang University , Hangzhou , 310027 , China . Email: rtang@zju.edu.cn; b Department of Virology , State Key Laboratory of Pathogen and Biosecurity , Beijing Institute of Microbiology and Epidemiology , Beijing , 100071 , China . Email: qincf@bmi.ac.cn; c Center for Biomaterials and Biopathways , Department of Chemistry , Zhejiang University , Hangzhou , 310027 , China; d State Key Laboratory of Silicon Materials , Zhejiang University , Hangzhou , 310027 , China

## Abstract

Exploring formulations that can improve the thermostability and immunogenicity of vaccines holds great promise in advancing the efficacy of vaccination to combat infectious diseases. Inspired by biomineralized core–shell structures in nature, we suggest a polyethyleneimine (PEI)–silica–PEI hybrid coated vaccine formulation to improve both thermostability and immunogenicity. Through electrostatic adsorption, *in situ* silicification and capping treatment, a hybrid coating of silica and PEI was assembled around a vaccine to produce vaccine@PEI–silica structures. Both *in vitro* and *in vivo* experiments demonstrated that the thermostability and immunogenicity of the modified vaccine were significantly improved. The modified vaccine could be used efficiently after long-term exposure at room temperature, which would facilitate vaccine transport and storage without a cold chain. Furthermore, mechanistic studies revealed that the PEI–silica–PEI coating acted as a physiochemical anchor as well as a mobility-restricting hydration layer to stabilize the enclosed vaccine. This achievement demonstrates a biomimetic surface-modification-based strategy to confer desired properties on biological products.

## Abstract 2

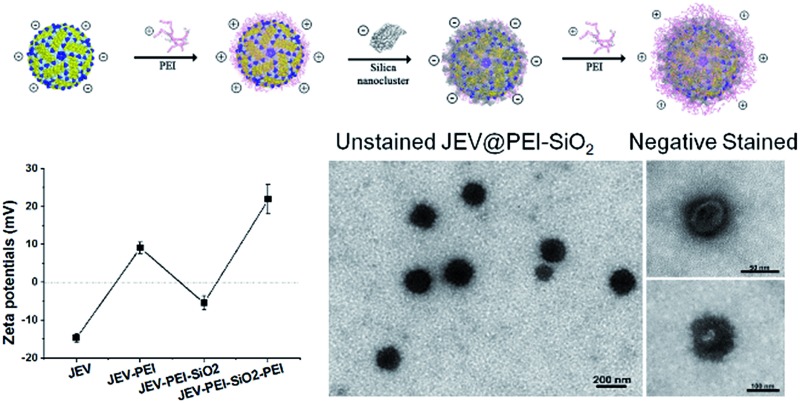
Inspired by biomineralized core–shell structures, we suggest a polyethyleneimine–silica hybrid coated vaccine formulation to improve both thermostability and immunogenicity.

## Introduction

Vaccines, especially live-attenuated vaccines for pathogens, are highly sensitive to temperature shifts, thus requiring continuous refrigeration to maintain their potency.^1^ Unfortunately, poor regions suffering from present vaccine-preventable infectious diseases are lacking extensive and reliable refrigeration facilities,^2,3^ and the deviation of vaccines from a cold chain during storage and delivery is unavoidable, resulting in the waste of a large proportion of vaccines.^4^ Therefore, the thermal instability of vaccines has become a significant obstacle to global vaccination programs and viral-based therapy.^5^ Robust vaccine formulations with improved thermostability and immunogenicity hold great promise to address these limitations. Several approaches, such as the preparation of carbohydrate glasses, the addition of silk protein, and biomimetic mineralization have been used to prepare thermostable and efficient formulations.^6–10^ However, there are still no satisfactory formulations for most vaccines.

Natural organisms including ancient phages and some plants use biomolecules to deposit amorphous silica hybrid coatings to survive environmental stresses.^11–14^ Our recent studies have also demonstrated that exterior silica nanoclusters could improve the thermostability of yeast cells and picornaviruses.^15–17^ In addition, amorphous silica is commonly used as a food additive, and generally recognized as safe by the US Food and Drug Administration.^18^ These achievements indicate that an exterior silica layer can be used to develop thermostable vaccine formulations. However, the direct deposition of silica on enveloped viral vaccines without hampering their original efficacy is difficult to realize, because they lack silica-accumulating sites and are highly sensitive to pH-tuned modification. In nature, organisms use biomolecules that are rich in cationic amino acids or polyamines to direct biosilica deposition processes under physiological conditions.^19–21^ Polyethyleneimine (PEI), a polyamine that can be synthesized in linear or branched forms with various molecular weights, may represent an ideal nucleating and stabilizing agent to control the deposition of silica nanoclusters on vaccines, as it has been widely used as a gene transfection agent and mucosal adjuvant.^22,23^ Due to the ability of PEI to facilitate cargo delivery into cells expressing heparan sulphate proteoglycans (HSPG), such as antigen-presenting cells (APC), we herein hypothesize that a vaccine formulation with a PEI–silica hybrid coating may exhibit an improved immune-stimulating ability.

**Fig. 1:**
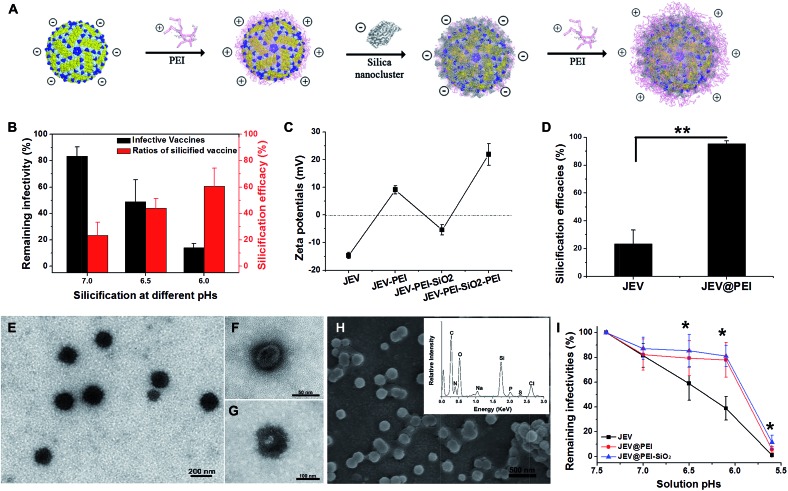
Polycationic molecule mediated *in situ* silicification of JEV. (A) Schematic illustration of the assembly of PEI–silica hybrid nanocoatings on the vaccine. (B) The sensitivity of JEV to pH-tuned silicification by adjusting solution pH to different acidities, with their remaining infectivity and silicification efficacies examined. (C) Zeta-potential of JEV, and JEV coated with PEI, PEI–SiO_2_ and PEI–SiO_2_–PEI sandwich layers. (D) The silicification efficacies of JEV at pH 7.0 with or without adding PEI as a nucleating agent. (E) TEM images of silicified vaccine JEV@PEI–SiO_2_ without any staining treatment. (F and G) TEM images of negatively stained JEV@PEI–SiO_2_, image (G) depicts silicified JEV that was almost totally encased by PEI–silica composites. (H) SEM images of JEV@PEI–SiO_2_ nanoparticles, inset represents EDX analysis of JEV@PEI–SiO_2_. (I) pH sensitivity of JEV, JEV@PEI and JEV@PEI–SiO_2_. (*P < 0.05, **P < 0.01, *n* ≥ 3, data represented as means ± SDs).

In the present study, using a clinically approved Japanese encephalitis vaccine (JEV) as a model, we propose a concept of designing a thermostable vaccine formulation, in which the vaccine surface is modified by PEI–silica–PEI sandwich coatings (Fig. 1A). As per our expectation, both *in vitro* and *in vivo* assessment results demonstrate that this hybrid material coated vaccine formulation not only exhibits significantly improved thermostability over a wide temperature range, but also enhances the potency of the vaccine to elicit humoral and cellular immune responses.

## Results

### Assembly of PEI–silica–PEI hybrid coatings on viral vaccine

As a typical enveloped viral vaccine, JEV SA-14-14-2 is highly acid labile. It is inactivated even when the solution pH is slightly below neutral. For example, treatment at pH = 5.6 for 30 min resulted in >95% loss in JEV vaccine titers (Fig. S1A†). However, the previously suggested pH-programmed silicification always needs an acidic procedure at a pH range of 5.5–6.0 to silicify the viral particles. Since this acidic treatment unavoidably leads to a significant decrease in JEV infectivity, pH-controlled silicification could not be directly used to silicify JEV without greatly reducing the original potency (Fig. 1B). Therefore, an alternative approach should be suggested to ensure the silification of this enveloped viral vaccine. Inspired by the previous achievements of biosilicification, silica-nucleating molecules, such as PEI and protamine, were introduced on the vaccine surface to realize efficient silicification under physiological conditions.^15,24–28^ Due to the negative zeta potential of the JEV vaccine particles, polycationic PEI molecules were adsorbed on the virion surface and acted as the nucleation sites for *in situ* silica deposition in a near-physiological environment (Fig. 1C). With these adsorbed cationic PEI molecules, the JEV vaccine could be efficiently silicified under neutral conditions without severely impairing its original potency (Fig. S1B†). When silica nanoclusters were attached to PEI-modified vaccine particles, the resulting hybrid of vaccine and mineral materials could readily be separated from solution by normal-speed centrifugation (16 000 g, 10 min) due to the relatively high gravity of silica. This feature enabled us to estimate the silicification efficacies of the vaccine by quantifying the viral particles in the supernatant and precipitate using plaque assays. It was found that more than 90% of PEI coated JEV vaccines (JEV@PEI) were efficiently silicified at neutral pH, whereas only 20% of native JEV vaccines could be silicified (Fig. 1D). The detrimental effect on vaccine infectivity caused by this PEI-mediated silicification was also evaluated and the results showed that >70% of initial vaccine titers were preserved in the presence of the PEI–silica hybrid coating (Fig. S1B†). Finally, 20 μg mL^–1^ of PEI molecules were added to the solution as a capping agent to stabilize the formed PEI–silica hybrid coating.

Under transmission electron microscopy (TEM), the silicified JEV@PEI vaccines could be directly observed and these dispersive nanoparticles had typical diameters of about 100 nm (Fig. 1E). The inner 50–60 nm virion was identified by negatively staining silicified JEV@PEI using phosphotungstic acid, which was surrounded by an uneven inorganic layer (Fig. 1F and G). Further scanning electron microscopy (SEM) confirmed the sphere-like structure of silicified JEV@PEI (Fig. 1H), and energy dispersive X-ray (EDX) diffraction analysis revealed that the modified outermost layers were mainly composed of C, N, O, Si, and S (Fig. 1H, inset), indicating the co-existence of organic and silica components. To verify the sandwich coating of the PEI–silica–PEI on virions, we carried out zeta-potential measurements after each treatment. As expected, the surface zeta-potentials changed alternately with silica nanoclusters or PEI as the outer layer (Fig. 1C). These results indicated the successful assembly of sandwich PEI–silica–PEI layers on the JEV vaccine and the new formulation was named JEV@PEI–SiO_2_.

Usually, viral vaccines cannot be separated by using a normal centrifugation method. A new characteristic of JEV@PEI–SiO_2_ was that the sandwich coating altered the surface electrostatic potentials of the viral particles from negative to positive (Fig. 1C), and increased their gravity density due to the attachment of the PEI–silica nanoclusters; therefore, JEV@PEI–SiO_2_ could be effectively separated and concentrated from solution by normal speed centrifugation (Fig. 1D). Besides, we found that the PEI–silica–PEI coating improved the acid resistance of the JEV vaccine at pHs ranging from 5.6–7.4 (Fig. 1I). These results showed that the assembly of the PEI–silica–PEI coating made JEV easier to separate and more robust.

### Biological activity and immunogenicity of PEI–silica coated vaccine

**Fig. 2:**
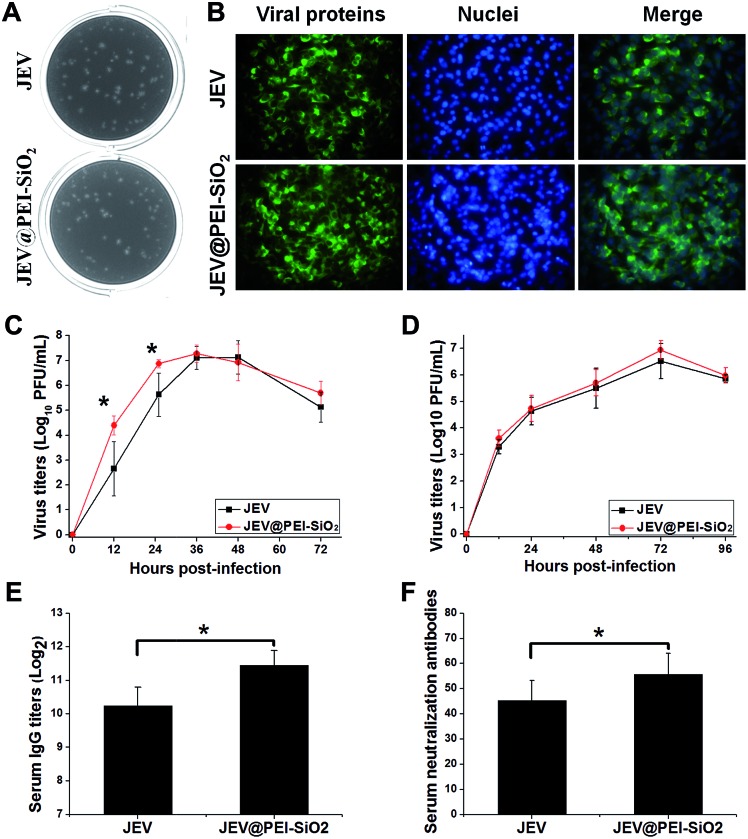
Biological activity and immunogenicity of JEV and the silicified vaccine JEV@PEI–SiO_2_. (A) Plaque morphologies in BHK21 cells. (B) The indirect immunofluorescence assays (IFA) of JEV and JEV@PEI–SiO_2_ in BHK21 cells at 36 h post-infection. (C) Growth curves of JEV and JEV–PEI–SiO_2_ in BHK21 cells, and (D) Vero cells (M.O.I. = 0.1). (E) The levels of serum IgG antibody and (F) neutralization antibody in mice immunized by the same amount of JEV or JEV@PEI–SiO_2_. (*P < 0.05, *n* ≥ 3, data represented as means ± SDs).

To study the availability of this formulation, we first examined the effect of PEI–silica hybrid coatings on the inherent biological properties of the vaccine. The results showed that both JEV@PEI and JEV@PEI–SiO_2_ still caused typical cytopathic effects in susceptible BHK-21 and Vero cells, and exhibited similar plaque morphologies to the native ones (Fig. 2A and S2A†) when diluted in a non-silica solution. And the infection of JEV@PEI or JEV@PEI–SiO_2_ caused efficient viral antigen expression in the cytoplasm of host cells (Fig. 2B and S2B†). Further examinations of their growth curves showed that JEV@PEI–SiO_2_ had similar growth patterns to JEV in BHK-21 cells (Fig. 2C) and Vero cells (Fig. 2D), but the vaccine titers produced in JEV@PEI–SiO_2_ infected cells were slightly higher than those of native JEV in the early stage (Fig. 2C). The conferred enhancement of infection can be explained by the interaction of positively charged PEI with cell surface HSPG, which increased the efficiency of viral attachment and the entry into host cells.

Then, mice were subcutaneously immunized with JEV and JEV@PEI–SiO_2_ to evaluate and compare the immunization potency. The *in vivo* examinations showed that JEV@PEI–SiO_2_ induced 2-fold higher serum IgG antibody titers (Fig. 2E) and slightly higher levels of the neutralization antibody (Fig. 2F), in comparison with those elicited by native JEV. These results indicated that the assembled PEI–silica hybrid coatings of JEV@PEI–SiO_2_ moderately improved the immunogenicity of the vaccine.

### Thermal stability of PEI–silica–PEI coated vaccine

**Fig. 3:**
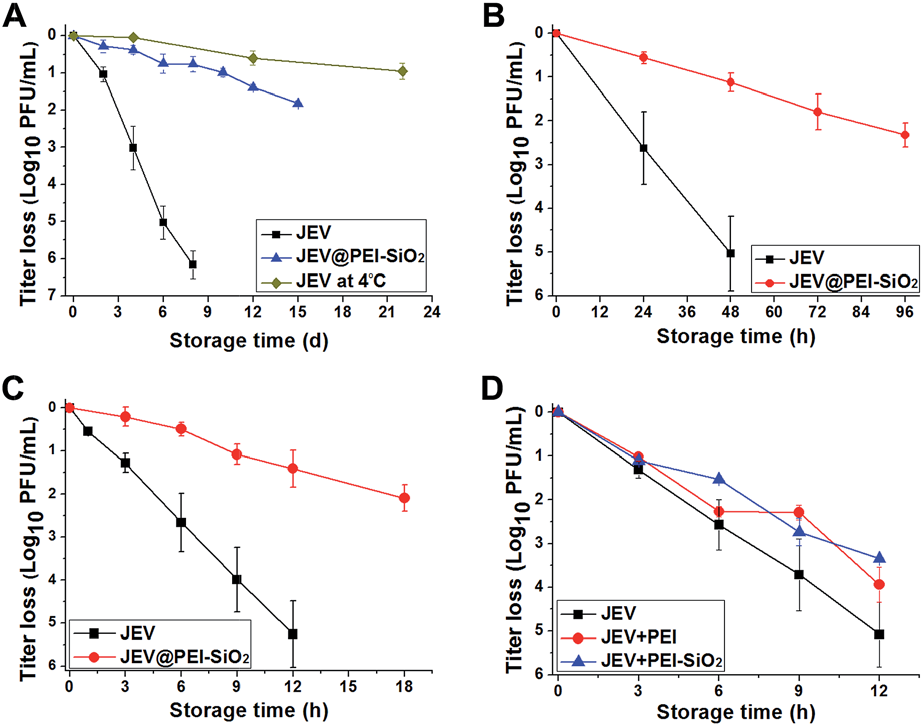
Thermostabilities of native JEV, JEV@PEI and JEV@PEI–SiO_2_ in liquid form. (A) Thermal-inactivation curves of JEV in native and JEV@PEI–SiO_2_ formulations at 25 °C, or (B) 37 °C, or (C) 42 °C, with the thermal-inactivation curves of native JEV at 4 °C as a reference. (D) Thermal-inactivation curves of JEV, JEV@PEI, and JEV mixed with *ex situ* synthesized PEI–silica composites at 42 °C. (*n* ≥ 4, data represented as means ± SDs).

We evaluated the thermal degradation rates of silicified JEV in different formulations, and found that vaccines in the JEV@PEI–SiO_2_ formulation exhibited the most desirable thermostability (Fig. S3†). Further examination revealed that the PEI–silica–PEI coatings significantly improved the thermostability of JEV at a wide range of temperatures (Fig. 3A–C). At a temperature of 25 °C, native JEV lost approximately 1 log_10_ PFU titer within 2 days and was almost totally inactivated after 1 week. The thermal stability of JEV was effectively improved by PEI–silica–PEI coatings. It took 12 days for JEV@PEI–SiO_2_ to loose approximately 1 log_10_ PFU titer at 25 °C. This activity loss tendency at room temperature was only slightly higher than that of JEV at 4 °C (Fig. 3A). At higher temperatures of 37 °C (Fig. 3B) and 42 °C (Fig. 3C), JEV@PEI–SiO_2_ could retain 90% of its original infectivity after storage times of >48 hours and ∼10 hours, respectively. Generally, the thermal inactivation rate of the vaccine in the JEV@PEI–SiO_2_ formulation was almost 5-fold slower than that of the native formulations. Clearly, the thermostability of the enveloped JEV vaccine could be significantly improved in the JEV@PEI–SiO_2_ formulation.

The thermal protective effect of adsorbed PEI and an *ex situ* synthesized PEI–silica nanocomposite was also studied. The thermal inactivation assays at 42 °C showed that the thermostability of JEV@PEI and JEV mixed with the PEI–silica nanocomposite was only slightly better than unmodified JEV (Fig. 3D), implying that the anchoring of PEI–silica nanoclusters on the vaccine initiated by *in situ* silicification was important for exerting their remarkable thermal protective role. Therefore, it is the *in situ* rather than *ex situ* treatment that is the key to guaranteeing the thermostability improvement using silica materials.

### *In vivo* examinations of PEI–silica–PEI coated vaccine

**Fig. 4:**
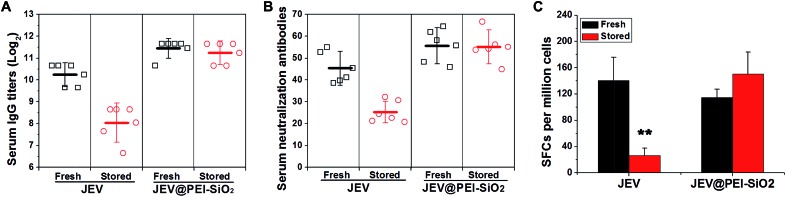
Animal experiments with fresh or stored JEV and JEV@PEI–SiO_2_. (A) The levels of elicited serum IgG antibody and (B) serum neutralization antibody in mice immunized by fresh or 18 day-stored (25 °C) JEV and JEV@PEI–SiO_2_. (C) The frequencies of JEV-specific IFN-γ secreting splenocytes of mice 12 days post-immunization were determined by quantifying the numbers of spot-forming cells (SFCs) with an ELIspot assay.

Mice were immunized with native JEV and JEV in the formulation of JEV@PEI–SiO_2_ after 18 days' storage at 25 °C, to confirm the efficacy and thermostability. In one-shot vaccination, the levels of serum IgG antibody (Fig. 4A) and neutralization antibody (Fig. 4B) elicited by stored JEV vaccines were decreased by more than 4-fold and 2-fold respectively. However, there was only a negligible decrease in the levels of JEV@PEI–SiO_2_ elicited serum lgG antibody and neutralization antibody after the same treatment, and they were still similar to that elicited by fresh JEV vaccines (Fig. 4A and B). In prime–boost vaccination, the levels of IgG antibody and neutralization antibody elicited by 18 day stored JEV decreased by >8-fold, whereas only a slight decrease in JEV@PEI–SiO_2_ induced neutralization antibodies occurred after the same storage (Fig. S4†).

Additionally, the potency of the JEV@PEI–SiO_2_ vaccine formulation to elicit a cellular response was evaluated by measuring JEV-specific IFN-γ secreting splenocytes of immunized mice with ELIspot assays.^29,30^ After storage for 18 days at room temperature, the frequencies of IFN-γ secreting splenocytes elicited by stored JEV vaccines decreased significantly, >80% lower than that elicited by fresh JEV (Fig. 4C). However, stored JEV@PEI–SiO_2_ still induced high frequencies of IFN-γ secreting cells in mice splenocytes, similar to those elicited by fresh JEV and JEV@PEI–SiO_2_. It follows that long-term storage of enveloped viral vaccines at ambient temperature could be achieved by assembling exterior PEI–silica–PEI coatings.

### Protection mechanisms of exterior organic–silica nanocomposites

**Fig. 5:**
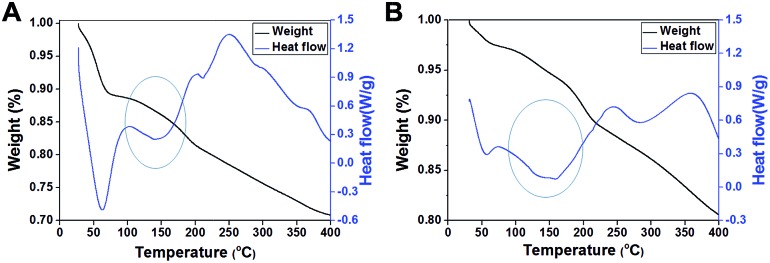
TGA-DSC analyses of freeze-dried powders of (A) native JEV and (B) JEV@PEI–SiO_2_. The results revealed that almost all water molecules in native vaccines were deprived at 60 °C, whereas most water molecules in JEV@PEI–SiO_2_ were not lost until the temperature reached 150 °C, indicating that the PEI–silica composite had an excellent water confining effect.

According to a previous study,^16^ we speculated that exterior PEI–silica hybrid nanocoatings could confine a large amount of water molecules by forming hydrogen bonds,^31^ thus acting as a molecule mobility buffering layer, as well as a physically confining matrix to prevent the inner vaccine undergoing conformational changes.^16,32^ We verified this hypothesis by examining the weight loss of samples and the heat-flow with temperature increase using thermogravimetric analysis (TGA) and differential scanning calorimetry (DSC). Most water molecules in the native sample were deprived before 100 °C (Fig. 5A), whereas most of the water molecules in silicified JEV@PEI–SiO_2_ vaccines were not deprived until the temperature reached 150 °C according to their weight loss tendency (1.5%) and heat adsorption peak (Fig. 5B). These results indicated that a large amount of water molecules in JEV@PEI–SiO_2_ was in a hydrated form, and that the hydrogen bonds with PEI–silica nanocomposites needed to be broken before their deprivation. Combining these results, we rationally suggested that the organic–inorganic hybrid nanocoatings and nearby water molecules constituted a protective hydration layer around the vaccines, which could relieve the destructive bond exchange between the aqueous solution and vaccines by decreasing the thermal motion transfer.

## Discussion

In our recent study, we illustrated the potential application of *in situ* silicification in stabilizing non-enveloped picornaviruses.^16^ However, the pH-tuned silicification approach cannot be directly used to silicify enveloped viral vaccines because they are highly sensitive to changes of solution pH. Effective nucleating and flocculating agents are required to realize their efficient silicification under physiological conditions. Due to the abundance of amine groups and the adjuvanticity of polycationic PEI, it was introduced onto the surface of the vaccine by electrostatic adsorption to induce the *in situ* deposition of silica nanoclusters. Furthermore, PEI molecules are added as a capping agent to stabilize the formed silica nanoclusters, producing a dually functional sandwich coating of PEI–silica–PEI for JEV to fulfil thermal protective and immune-stimulating roles.

It is worth noting that PEI can be replaced by a natural cationic polypeptide, low molecular weight protamine, to facilitate the efficient silicification of the JEV vaccine under near-physiological conditions (Fig. S5†) without obviously compromising its native biological activities (Fig. S6†). This means that we can choose either polymers rich in amines or functional polypeptides to assemble a desired organic–silica hybrid coating on a vaccine, following a general principle of silicification regulation using cationic amine residues. Similar to the PEI–silica–PEI coating, *in situ* synthesized protamine–silica–protamine coatings also significantly improved the thermostability of vaccines (Fig. S7†). However, the thermostability improvements of the vaccine conferred by the PEI–silica–PEI sandwich coating are better than those conferred by the protamine–silica–protamine coating (Fig. 3 and S7†), which may be due to the better water confining effect of branched PEI macromolecules.

Additionally, the PEI–silica–PEI hybrid coatings also improve the immunogenicity of JEV vaccines, facilitating them to induce a robust humoral and cellular response even after long-term storage. In this vaccine formulation, we suggest that the anchored polycationic PEI molecules can facilitate an interaction between vaccines and APCs, thus promoting their uptake and processing by immune cells. Notably, organic–silica nanocoatings produced by protamine or linear PEI fail to lead to such an obvious improvement of the vaccine immunogenicity (Fig. S8 and S9†), indicating that the use of branched PEI molecules may be a better choice.

Previously, we have proposed the use of calcium phosphate, an inorganic adjuvant, to coat viral vaccines to improve both their thermostability and immunogenicity, but the thermostability improvements conferred by calcium phosphate are relatively low.^10^ The synthesized calcium phosphate cannot be stabilized well in aqueous solution for a long time due to its biological degradable profile, which also limits its application. By introducing a hydrated silica exterior, we significantly improved the thermostability of the viral vaccines, with no enhancement of immunogenicity. In the present study, by taking advantage of the excellent water confining effect of amorphous silica, and the nucleation ability and adjuvanticity of PEI polymer, we successfully assembled a thermally protective but immune-stimulating PEI–silica–PEI hybrid coating for a viral vaccine, through coating the vaccine with PEI, *in situ* silicification, and a subsequent capping treatment. Some of the literature has shown that synthetic silica and PEI have good biocompatibility, and they have been studied as vaccine delivery vehicles and adjuvants.^18,33^ A single dose of vaccination with <20 μg PEI and <1 mg silicates can be considered as safe.^22,34^ In our case, the introduced amounts of both PEI and silicates can be controlled around these limits to support the potential use of this vaccine formulation. Therefore, silicified vaccine formulations could be used with a guarantee of material safety.

Actually, this idea comes from organisms that accumulate silica on their cell walls to improve thermal tolerance.^35^ Analogously, the exterior organic–silica hybrid nanocoatings around the vaccine can improve its thermostability. Our study may provide a good example of using a nature-evolved strategy to produce a robust vaccine formulation. This vaccine formulation combined the adjuvant PEI molecule, thermally protective silica and the vaccine together, and will guarantee vaccine efficacy without any access to refrigeration for several days. Such an achievement can efficiently advance the terminal distribution of vaccines from local storage centres to immunization clinics or mobile on-site utilization, and extend immunity against deadly infectious diseases to the world’s poorest areas.

## Conclusions

By combining layer-by-layer and *in situ* silicification treatment, a PEI–silica hybrid nanocoating was assembled on an enveloped viral vaccine under near-physiological conditions, producing a new robust formulation. Taking advantage of silica’s nucleating ability, the cell transfection ability of PEI, and the hydration ability of amorphous silica, the vaccine formulation exhibited significantly improved thermostability and immunogenicity, and it still induced a high level humoral and cellular immune response after long-term storage at ambient temperature. The current biomimetic approach suggests a cost-effective, material-based vaccine formulation to advance cold chain independent vaccine distribution.

## Experimental section

### Biomimetic silicification of JEV vaccines

Fresh silicic acid (30 mM) was prepared fresh before use as follows: 5.3 μL of sodium silicate (Sigma) was diluted in 1 mL of PBS (pH 7.4); 20 μL of 1.25 M HCl was then added to adjust the solution to pH 7.5–8.0. To obtain *in situ* silicified JEV vaccine, the cationic molecules protamine or 600 Da/10 kD polyethyleneimine (PEI) were added into vaccine solutions (∼10^7^ PFU mL^–1^) at a final concentration of 100 μg mL^–1^ and incubated at 37 °C for 5 min, and then 30 mM fresh silicic acid was added to reach a final concentration of 3 mM. The solution pH was adjusted to 6.5–7.0 with HCl to initiate silicification, reacting for 15–30 min. Finally, 20 μg mL^–1^ protamine or 600 Da/10 kD PEI was added into the silicified vaccine solution as a capping agent.

### Thermal stability tests

Native JEV vaccines, JEV@PTM, JEV@PEI, JEV@PEI–SiO_2_, JEV@PTM–SiO_2_, and JEV mixtures with *ex situ* synthesized PTM–SiO_2_/PEI–SiO_2_ were stored at 4 °C, 25 °C, 37 °C, and 42 °C, and the samples were collected periodically. Their remaining infectivity was determined by plaque assays. The remaining percentages of infectivity were calculated and represented on a logarithmic scale *vs.* incubation time (*n* ≥ 4); the data are represented as means ± standard deviations.

### Mouse experiments

Mice experiments were approved by and performed in strict accordance with the guidelines of the Animal Experiment Committee of the Beijing Institute of Microbiology and Epidemiology (China). 4 week old BALB/c mice were subcutaneously immunized with fresh or stored JEV, JEV@PTM–SiO_2_, and JEV@PEI–SiO_2_ with the same initial titers before storage (200 μL, 10^7^ PFU mL^–1^). In prime–boost vaccination, the immunized mice were further boosted with fresh/stored JEV and JEV@PEI–SiO_2_ at 4 weeks post 1st inoculation. Mice sera were collected at 2 and 4 weeks post-injection. The levels of serum IgG antibody and neutralization antibody were detected using enzyme-linked immunosorbent assay (ELISA) and standard plaque reduction neutralization tests (PRNT), respectively.

### Standard plaque reduction neutralization tests

Mouse serum was serially 2-fold diluted in DMEM, starting at 1 : 8. Virus suspensions (150 μL at 100 PFU) were mixed with 150 μL of diluted sera, and the mixtures were incubated at 37 °C for 1–1.5 h. The mixtures were then added to 90–100% confluent BHK-21 cells and incubated for 1 h. Then the cells were washed and incubated with DMEM supplemented with 2% FBS and 1% low melting point agarose, and cultured at 37 °C, 5% CO_2_ for about 3 days before fixation with 4% formaldehyde. Endpoint titers were calculated according to the Karber method, as previously described.^16^
