# Supplementary material for: Robust vaccine formulation produced by assembling a hybrid coating of polyethyleneimine–silica
Source: Chem Sci. 2015 Dec 10;7(3):1753–9. doi: 10.1039/c5sc03847b (PMC5592373; doi:10.1039/c5sc03847b)
Supplement: Supplementary file 1 [file SC-007-C5SC03847B-s001.pdf]

Electronic Supplementary Material (ESI) for Chemical Science.
This journal is © The Royal Society of Chemistry 2015

Supporting Information

Robust vaccine formulation produced by assembling hybrid coating of polyethyleneimine-silica

Guangchuan Wang,[a,b] Hangyu Zhou,[c] Qing-Gong Nian,[b] Yuling Yang,[c] Cheng-Feng Qin,*[b] and Ruikang Tang*[a,c,d]

[a.]Qiushi Academy for Advanced Studies, Zhejiang University, Hangzhou, 310027, China.

[b.]Department of Virology, State Key Laboratory of Pathogen and Biosecurity, Beijing Institute of Microbiology and Epidemiology, Beijing, 100071, China.

[c.]Center for Biomaterials and Biopathways, Department of Chemistry, Zhejiang University, Hangzhou, 310027, China.

[d.]State Key Laboratory of Silicon Materials, Zhejiang University, Hangzhou, 310027, China.

*Correspondence authors. Email: rtang@zju.edu.cn or qincf@bmi.ac.cn

Materials and Methods

Cells and viruses. A live-attenuated vaccine strain SA14-14-2 of Japanese encephalitis virus (JEV) vaccine was kindly provided by Chengdu Institute of Biological Products (China). Baby hamster kidney (BHK-21) cells, and African green monkey kidney (Vero) cells were obtained from the American Type Culture Collection (ATCC) and were cultured in DMEM media supplemented with 8% fetal bovine serum (FBS) at 37°C and 5% CO_2. All virus stocks were prepared in BHK-21 cells and were collected at 36-48 h post-infection when typical cytopathic effects developed; the stocks were titrated in BHK-21 cells and stored at −70°C until use.

Indirect immunofluorescence assays (IFA). BHK-21 cells in 48-well plate at 90%-100% confluence were infected with JEV, silicified JEV vaccines JEV@PTM-SiO_2 and JEV@PEI-SiO_2 at a multiplicity of infection (M.O.I.) of 0.1. 24-36 h post-infection, the infected cells were fixed with pre-cooled acetone at -20°C for 30 min. And then the cells were washed with PBS and incubated with primary JEV-specific polyclonal antibodies for 1 h, followed by three times washing with PBS and the incubation with Alexa Fluor 488-conjugated goat anti-mouse IgG secondary antibody for 45 min.

DAPI was then added, and the cells were incubated at room temperature for 5 min to stain the nuclei. Fluorescence was detected after PBS washing for three times.

Plaque assays. BHK21 cells at 80%-90% confluence in 12-well plates were incubated with 333 μL of serially diluted viral vaccines. After adsorption for 1-2 h, the cells were washed and incubated with DMEM containing 2% FBS and 1% low melting point agarose for 3 days. The infected cells were fixed with 4% formaldehyde and stained using a crystal violet solution (1% crystal violet, 0.85% NaCl, and 2% formaldehyde).

Zeta-potentials. The zeta-potentials of PEI, PEI-silica and PEI-silica-PEI coated JEV particles were measured by using a Zetasizer Nano ZS equipment (Malvern Instruments, UK). All of the samples were diluted in PBS (pH 7.4, 10 mM), and the measurements were performed at room temperature with more than 3 repeats.

Growth curves. Growth curves of native and silicified JEV vaccines in BHK-21 cells were determined by infecting monolayer BHK-21 cells (90%-100% confluence) with JEV, JEV@PTM-SiO_2, JEV@PEI-SiO_2 at an M.O.I of 0.1. After 1-2 h of adsorption, the cells were washed and incubated with DMEM containing 2% FBS. Samples were collected at intervals, and their titers were examined by plaque assays.

Electron microscopy. Solutions containing native or silicified JEV vaccines JEV@PEI-SiO_2, JEV@PTM-SiO_2 were dropped onto carbon-coated copper TEM grids (400 meshes, Agar Scientific). The samples were dried at room temperature before observation. TEM observations were performed before and after negative staining with phosphotungstic using a JEM-1200EX instrument (JEOL, Japan). SEM and EDX analyses were performed using an S-4800 instrument (HITACHI, Japan).

Acid sensitivity tests. To examine the acid sensitivity of JEV vaccines, about 2×10^6 PFU virus in 100 μL DMEM was mixed with 300 μL of 50 mM phosphate-buffer saline (PBS) solutions of different pHs, and incubated for 30 min at room temperature. Then 100 μL of 1 M Tris (pH 7.4) was added to neutralize the solution, and the remaining infective viral particles in each sample were determined by plaque assays.

Enzyme-linked immunosorbent assay (ELISA). Serum IgG antibodies against JEV were detected using indirect ELISAs in 96-well flat-bottomed plates (Costar). The plates were coated with 1:50 diluted JEV vaccines in 0.1 M carbonate/bicarbonate buffer (pH 9.6), and incubated at 4°C overnight. After blocking with 2% bovine serum albumin (BSA) in PBS and twice washing with PBST, the

plates were incubated with serially diluted mice sera in duplicate wells for 1 h at 37°C. Then the plates were washed three times with PBST and incubated with 1:5000 diluted Peroxidase-conjugated horse anti-mouse IgG in PBS at 37°C for 0.5 h. After three times washing with PBST, TMB substrate was added. The absorbance of the plates was determined at 492 nm and corrected for background using PBS as control.

ELIspot assays. IFN-γ ELIspot mouse kits (BD Biosciences) were used according to the manufacturer's instructions. Briefly, 96-well filtration plates were coated overnight at 4°C with an IFN-γ capture monoclonal antibody; the plates were then washed and blocked with RPMI-1640 medium supplemented with 10% FBS for 2 h at room temperature. Splenocytes of immunized 12 days after infection were isolated and suspended in RPMI-1640 containing 10% FBS. Then 10^5 cells were then added into the plate, followed by the addition of JEV as the stimulation antigen. The plates were cultured overnight at 37°C and 5% CO_2. After washing once with water and three times with PBST, the plates were incubated with biotinylated IFN-γ detection antibody at room temperature for 2 h and then washed three times with PBST, after which the plates were incubated with HRP-conjugated streptavidin at room temperature for 1 h. Spots were revealed using an AEC substrate reagent kit (BD Bioscience) at room temperature and counted using an Immunospot Reader (Cellular Technology).

Statistical analyses. The statistical significance among different groups was analyzed using Student's *t*-test as implemented in SPSS software. The results with error bars are expressed as means ± standard deviations.

Supporting Figures

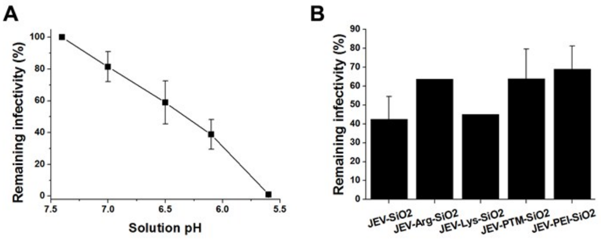

Figure S1. (A) pH sensitivity of JEV was determined by treating the viral particles with different acidic buffers for 30 min; the remaining infectivity was calculated as the percentage of their original ones. (B) Detrimental effect of different coating methods on the infectivity of JEV vaccines.

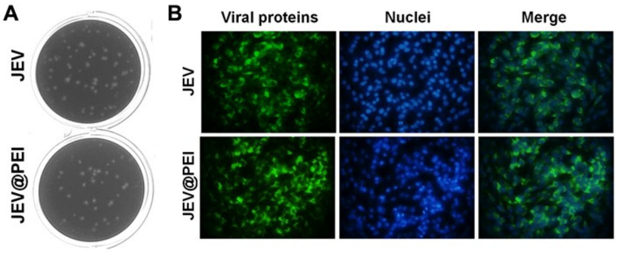

Figure S2. Biological properties of native JEV and JEV@PEI. (A) Plaque morphologies in BHK-21 cells. (B) The indirect immunofluorescence assays (IFA) to show viral protein expression in BHK-21 cells at 36 h post-infection.

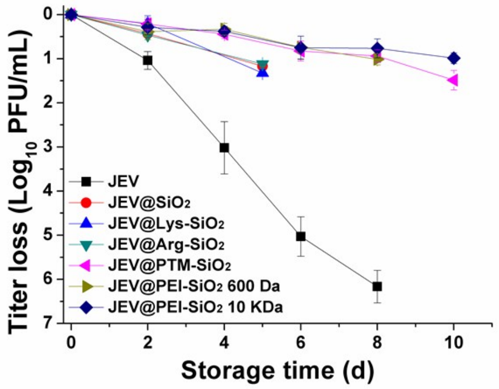

Figure S3. The thermal protective effect of silicified hybrid coating produced using different silicification approaches.

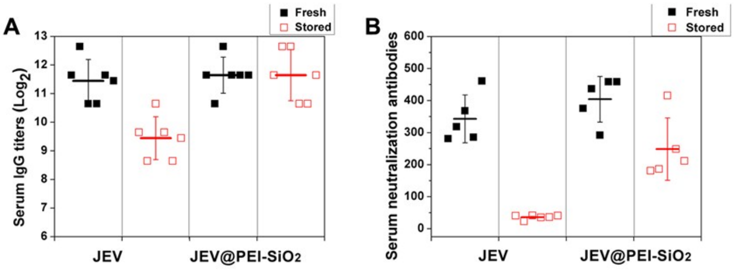

Figure S4. Animal practices by immunizing mice twice with fresh or 18-day-stored (25°C) stored JEV and JEV@PEI-SiO_2 with the same initial titers. (A) Elicited titers of serum IgG antibody. (B) Neutralization antibody in mice at 4 weeks post immunization.

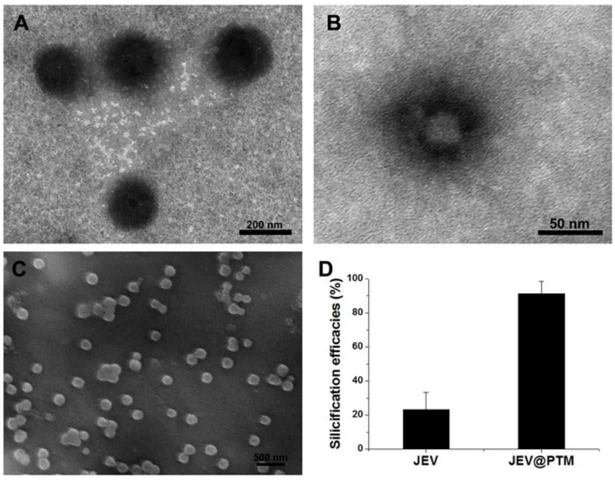

Figure S5. (A) TEM images of silicified JEV vaccine in formulation of JEV@PTM-SiO_2, inset represents TEM image at low magnification. (B) JEV@PTM-SiO_2 negatively stained with phosphotungstic, which revealed that silicified JEV that was partially or totally encased in protamine-silica nanocomposites. (C) SEM images of silicified vaccines in formulation of JEV@PTM-SiO_2.

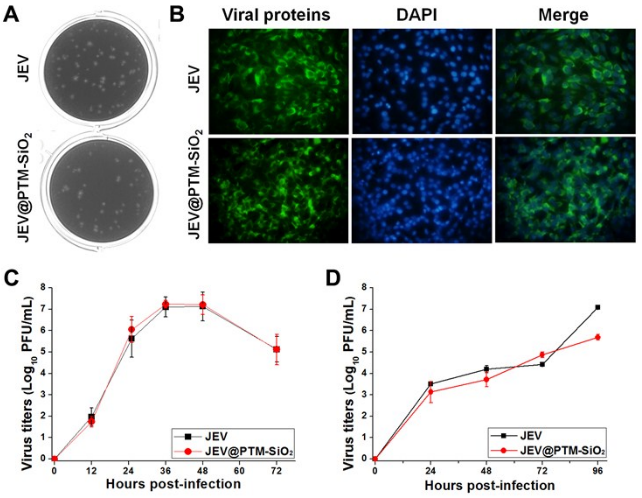

Figure S6. Biological characteristics of native JEV and silicified JEV@PTM-SiO_2. (A) Plaque morphologies in BHK-21 cells. (B) The indirect immunofluorescence assays (IFA) of JEV and JEV@PTM-SiO_2 in BHK-21 cells at 36 h post-infection. (C) Growth curves of JEV and JEV-PTM-SiO_2 in BHK-21 cells and (D) Vero cells. (M.O.I.=0.1; n≥3, data represented as means ± SDs).

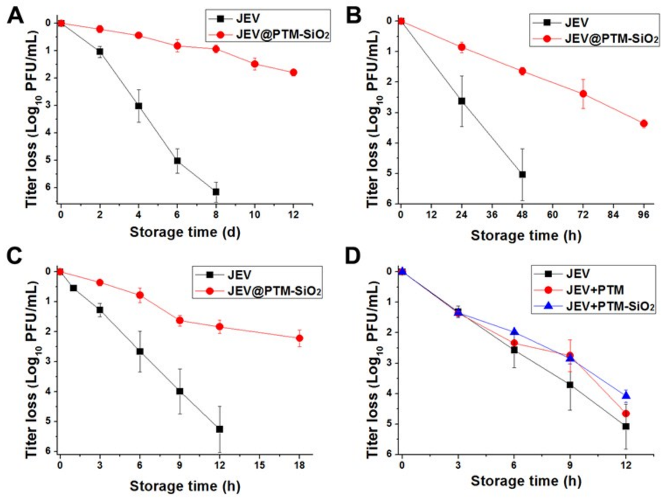

Figure S7. The thermostability of native JEV, Protamine coated JEV (JEV@PTM) and silicified JEV vaccines in JEV@PTM-SiO_2 formulation. (A) Thermal-inactivation curves of JEV vaccines in their native and JEV@PTM-SiO_2 formulations at 25°C, or (B) 37°C, or (C) 42°C. (D) Thermal-inativation curves of JEV vaccines, JEV@PEI formulations, or JEV complexed with *ex situ* synthesized PEI-silica composites (JEV+PTM-SiO_2) at 42°C. (n≥4, data represented as means ± SDs).

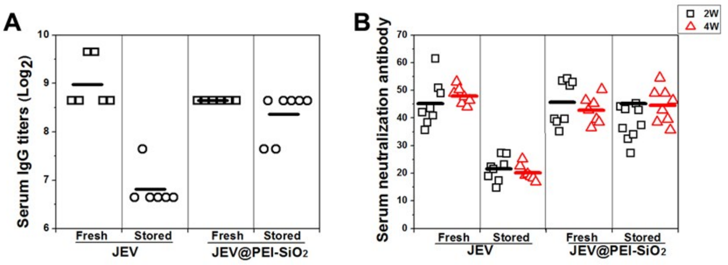

Figure S8. Animal practices with fresh prepared or 12-day (25ºC) stored JEV and JEV@PTM-SiO$_2$. (A) The titers of serum IgG and (B) neutralization antibody at 4 weeks post immunization.

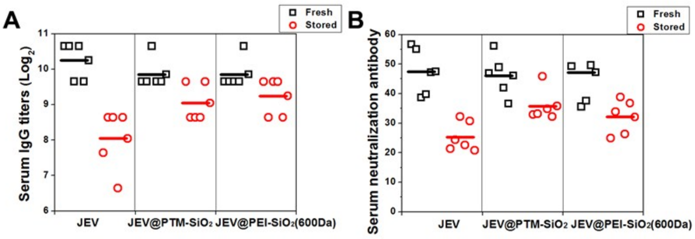

Figure S9. Animal practices with fresh prepared or 18-day (25ºC) stored JEV, JEV@PTM-SiO$_2$ and JEV@PEI-SiO$_2$ (linear PEI, Mw=600 Dalton); (A) the elicited titers of serum IgG and (B) neutralization antibody at 4 weeks post immunization.
